# Physiological and Transcriptomic Response of Grey Poplar (*Populus ×canescens* Aiton Sm.) to Cadmium Stress

**DOI:** 10.3390/plants9111485

**Published:** 2020-11-04

**Authors:** Martina Komárková, Jakub Chromý, Eva Pokorná, Petr Soudek, Pavlína Máchová

**Affiliations:** 1 Forestry and Game Management Research Institute, Strnady, 25202 Jiloviste, Czech Republic; chromy@vulhm.cz (J.C.); pokorna@vulhm.cz (E.P.); machova@vulhm.cz (P.M.); 2 The Czech Academy of Sciences, Institute of Experimental Botany, 16502 Prague, Czech Republic; soudek@ueb.cas.cz

**Keywords:** cadmium, gene expression, grey poplar, microsatellite analysis, mineral uptake, translocation factor

## Abstract

(1) Background: *Populus ×canescens* (Aiton) Sm. is a fast-growing woody plant belonging to the family *Salicaceae*. Two poplar genotypes characterized by unique phenotypic traits (TP11 and TP20) were chosen to be characterized and tested for a physiological and transcriptomic response to Cd stress. (2) Methods: A comparative analysis of the effects of exposure to high cadmium (Cd) concentrations (10 µM and 100 µM) of TP11 and TP20 was performed. (3) Results: Neither of the tested Cd concentration negatively affected plant growth; however, the chlorophyll content significantly decreased. The potassium (K) content was higher in the shoots than in the roots. The magnesium concentrations were only slightly affected by Cd treatment. The zinc content in the shoots of TP20 was lower than that in the shoots of TP11. Cd accumulation was higher in the roots than in the shoots. After 10 days of exposure, 10 µM Cd resulted in comparable amounts of Cd in the roots and shoots of TP20. The most significant change in transcript amount was observed in endochitinase 2, 12-oxophytodienoate reductase 1 and phi classglutathione S-transferase. (4) Conclusions: Our study provided new insights for effective assessing the ability of different poplar genotypes to tolerate Cd stress and underlying Cd tolerance.

## 1. Introduction

Environment plays a crucial role in ensuring global health. Different sources of environmental stress lead to severe problems in many parts of the world, including social and economic impacts. In recent years, soil heavy metal pollution has attracted global attention. Heavy metals tend to accumulate in living organisms and cause serious deleterious effects. In soil, cadmium (Cd) originates mostly as an emission from industrial processes. Due to its low adsorption coefficient and high soil-plant mobility, Cd may enter the food chain. Plant Cd uptake occurs mainly via roots and is taken up into the cell by passive and active pathways, mediated by carriers and channels permeable to essential nutrients [1,2,3]. Within plants, Cd causes phytotoxicity by inhibiting plant growth and respiration [4], causing the dysfunction of photosynthesis and stomatal closure [5], disrupting ATPase activity [6] or decreasing water and nutrient uptake and transport [7]. At the cellular level, high Cd concentrations can cause the overproduction of reactive oxygen species (ROS), protein oxidation, membrane lipid peroxidation [8], chromosomal aberration and DNA or RNA damage [9]. In addition, transcriptomic changes in several kinds of genes upon Cd stress have been investigated in several plant species [10,11].

The ability of plants to uptake and store heavy metals from soil into their parts can be used as an alternative technology called phytoremediation. Phytoremediation is a very promising method for the detection and removal of toxic metals accumulated in the environment [12]. This technique uses the ability of some plant species to remove contaminants by absorbing them into the aboveground plant parts. To date, many hundreds of different plant species that specialize in the hyperaccumulation of a particular contaminant have been described [13]. The most studied hyperaccumulator is probably *Thlaspi caerulescens* J.Presl & C.Presl, with a marked ability to accumulate zinc (Zn) and Cd into its aboveground tissues [14,15,16]. Other identified hyperaccumulators have also been described, for example, *Alyssum* species with nickel (Ni) phytoextraction potential [17], the arsenic (As) hyperaccumulator fern *Pteris vittata* L. [18], and *Arabidopsis halleri* L. with Zn and slight Cd hyperaccumulation capacities [19]. Unfortunately, the limited biomass of herbaceous plants prevents their application for phytoremediation on a large scale. Woody plants, including some poplar genotypes, have been proposed for phytoremediation due to their rapid growth, deep root system and high Cd accumulation capacity [20].

The diploid genus *Populus* (poplar) is known to have high amounts of intraspecific variability and widespread hybridization between ecologically differentiated species [21]. *Populus ×canescens* Aiton Sm. (grey poplar) is a product of ongoing gene flow between its parents, *Populus alba* L. and *Populus tremula* L. [22]. For grey poplar, both sexual and clonal reproduction are common [23]. Thus, genetic analyses (simple sequence repeat (SSR)) can be used to differentiate between asexual and sexual propagation and between different clones [24,25]. Simple sequence repeats are tandem repeats of DNA sequences 1-6 base pairs (bp) in length and represent powerful tools in screening plant genomic DNA usable in taxonomy, genetic mapping, population genetics and genotoxicity tests [26,27,28].

To elucidate Cd distribution and physiological responses to heavy metal stress in fast-growing woody plant species, two poplar (*P. canescens*) genotypes with unique phenotypic traits were exposed to 0, 10 or 100 µM CdCl_2_. The objective of this experiment was to address the following concepts: (1) the effect of Cd on the growth and mineral uptake in genotypes TP11 and TP20; (2) whether the selected poplar genotypes exhibited phytoextraction potential towards Cd; and (3) whether Cd exposure causes changes in the gene expression level. The obtained results may not only help us to better understand the physiological and transcriptomic regulation mechanisms of woody plants towards Cd exposure but also provide guidelines for the selection of genotypes with high Cd tolerance and phytoremediation potential.

## 2. Results and Discussion

### 2.1. Microsatellite Analysis

Since poplar trees are known to produce root sprouts and thus generate clones of one individual [29], it was necessary to distinguish the genotypes of selected trees. Signs of differentiation are well detectable by genetic analyses with molecular markers, such as nuclear SSRs. To determine whether the selected poplar plants shared the same genotype, they were screened for variation at 15 nuclear SSR loci. The tested SSRs, except ORPM14, were sufficiently polymorphic to be used for the genetic structure analyses of grey poplar. A summary of the allele size of the SSRs analysed in source plants is presented in Table 1. The simple sequence repeats WPMS5, WPMS20, ORPM14, ORPM16, ORPM127 and ORPM312 presented homozygous individuals with one allele each in TP11. In the second poplar genotype, 8 homozygous alleles were observed in SSRs WPMS19, WPMS20, ORPM14, ORPM16, ORPM60, ORPM127, ORPM220 and ORPM312. The same allele sizes in both genotypes were obtained only by 8 SSRs (Table 1). These results of our genetic analysis show that the microsatellite regions of the two selected poplar trees were different.

### 2.2. Effect of Cd on Plant Growth and Chlorophyll Content

*Populus canescens* plantlets were exposed to two Cd concentrations (10 µM and 100 µM) that did not abolish growth. The responses of poplar clones TP11 and TP20 to Cd were first screened by visual observation. Cadmium toxicity is often accompanied by characteristic leaf symptoms, including chlorosis and necrosis [30,31,32]. After the addition of 100 µM Cd, only slight leaf chlorosis was observed, while other plants (control and 10 µM treatment) did not show any significant symptoms of toxicity (data not shown).

The effects of Cd treatments on the growth parameters and chlorophyll content of *P. canescens* are shown in Table 2. Neither tested Cd concentration negatively affected root length at either time point (2 and 10 days after Cd exposure). Interestingly, longer roots were measured for both grey poplar genotypes after 2 and also 10 days of 10 µM and 100 µM Cd treatments compared to the controls (Table 2). Relatively similar root weights were detected for TP11 (75.0 ± 3.6 and 85.3 ± 4.2 mg) and TP20 (71.3 ± 5.5 and 84.3 ± 5.5 mg) at 2 days after 10 µM and 100 µM Cd application in comparison to the significantly different shoot weights after 2 days of 10 µM and 100 µM Cd treatment, reaching 255.7 ± 49 mg and 226.0 ± 56 mg in TP11 and 157 ± 39 mg and 151.0 ± 45 mg TP20, respectively. We found that prolonged treatment with both Cd concentrations resulted in an increase in biomass, especially in root length and root weight. Previous studies have shown that Cd addition leads to the dramatic inhibition of root growth, probably due to the influence of the homeostasis of auxin and ROS [33]. On the other hand, Schützendübel et al. [34] showed that 5 µM Cd stimulated root growth in hydroponically grown grey poplar, which correlates with our findings. A concentration of 50 µM Cd resulted in growth inhibition within 48 h [34]. In our experiment, root growth was not affected for either genotype when treated with 100 µM Cd.

In general, the contents of chlorophyll *a* and *a + b* declined in both poplar genotypes with prolonged Cd treatment (both 10 µM and 100 µM Cd) compared to the control conditions (Table 2). In TP 11 and TP20, the chlorophyll *b* content was not significantly different between the control and Cd treatments. Similarly, in TP20, the chlorophyll *b* content was not significantly different between the control and Cd treatments. Cadmium is known to be responsible for chloroplast damage and interfering with chlorophyll biosynthesis, causing an inverse correlation between the chlorophyll content and Cd concentration [35,36].

### 2.3. Effect of Cd on K, Ca, Mg and Zn Uptake and Translocation

Table 3 shows the mineral content in the roots and shoots of the two selected genotypes of *P. canescens*. The potassium (K) contents were significantly lower in the roots than in the shoots in both genotypes at all tested concentrations of Cd. The root K content in the TP11 genotype significantly increased only after 10 days of 100 µM Cd treatment (20.35 g kg^−1^) compared to the control conditions (11.70 g kg^−1^). In the shoots, TP11 exhibited a reduction in the K content (23.57; 24.29 g kg^−1^) after 2 days under both Cd treatments compared to the control (30.19 g kg^−1^) conditions. In contrast, enhanced levels of the K content in the shoots were observed in TP20 after 10 days of Cd exposure. Potassium is one of the most important plant nutrients with high mobility and significant effects on different metabolic processes [37]. K application enhanced Cd tolerance in bean plants [38]. In our experiment, an increase in K was observed mainly in the roots, which is consistent with the results from *Trifolium repens* L. [39] or *Triticum aestivum* L. [40].

Calcium (Ca) and Cd compete for Ca^2+^ channels and intracellular Ca-binding proteins, thus influencing their contents [41]. In our experiment, Ca accumulation values were higher in the shoots than in the roots of all tested TP11 and TP20 plants. However, the Ca concentrations in the roots and shoots were, in most cases, not significantly changed after Cd exposure in either of the tested genotypes. Only in TP11 shoots after treatment with 100 μM Cd for 2 days was a significant increase in Ca (8.54 g kg^−1^) detected compared to the control condition (6.60 g kg^−1^).

Magnesium (Mg) and Ca exhibit the same inhibitory effect due to the competition of these divalent cations. In our study, Mg concentrations were slightly reduced in the roots of both genotypes after 10 days of 10 μM Cd treatment. On the other hand, TP20 accumulated higher amounts of Mg in roots exposed to 100 μM Cd for 2 days. Regarding the shoot Mg content, a slight change was observed only in TP11, with an enhanced Mg content after 10 days of 100 μM Cd treatment (2.64 g kg^−1^) compared to control conditions (1.73 g kg^−1^).

Zinc (Zn) concentrations were quite homogenous among the plant parts. The only difference was that both clones exhibited slightly higher amounts of Zn in the shoots after 10 days of Cd treatment compared to control conditions. The Zn content was lower in TP20 shoots than in TP11 shoots both in the 2- and 10-day controls and Cd-treated plants. According to Tkalec et al. [42], Cd exhibits a negative effect on Zn uptake in the leaves and roots. Cd can be taken up by the same transporters that are involved in Zn uptake and transport [43,44].

### 2.4. Cadmium Accumulation, Translocation and TF

**Figure 1:**
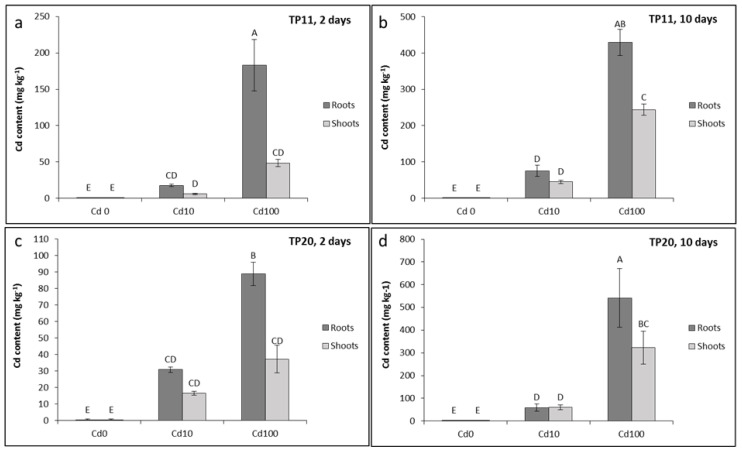
Content of Cd in the roots and shoots in *Populus ×canescens* (Aiton Sm.) genotypes TP11 (**a**,**b**) and TP20 (**c**,**d**) grown in perlite for 2 or 10 days with or without Cd. Data are presented as the mean ± SD (n = 3). Values followed by the same letter(s) are not significantly different according to Tukey’s test (*p* < 0.05).

To evaluate the ability of poplar to uptake Cd and accumulate it within the plant, the Cd content was analysed in the roots and shoots of the two selected grey poplar genotypes (TP11 and TP20). The translocation of Cd from the roots to the shoots was estimated by translocation factor (TF), which is defined as the ratio of the metal concentration in the shoot tissues to that in the root tissues. The concentration of Cd and Cd uptake was slightly different in the two tested *P. canescens* genotypes (Figure 1). With one exception (TP20, 10 days at 10 µM Cd incubation, Figure 1d), all plants accumulated more Cd in the roots than in the shoots. After 2 days of 100 µM Cd treatment, genotype TP11 accumulated significantly more Cd in the roots than genotype TP20 (Figure 1). In addition, the TF for genotype TP20 was higher in both Cd treatments (10 µM and 100 µM) and in both tested days compared to that for genotype TP11 (Table 4).

After 10 days of 10 µM Cd exposure, genotype TP20 accumulated comparable amounts of Cd in the roots (58.65 mg Cd kg^−1^) and in the shoots (59.62 mg Cd kg^−1^), which was reflected by a TF value greater than 1. The obtained results suggest variability among the grey poplar genotypes in Cd accumulation and translocation. Our data revealed that genotype TP20 may be a good candidate for Cd phytoextraction processes, similar to other tolerant poplar species, such as *P. alba* [45], *Populus deltoides* Marshall [46] or *Populus nigra* L. [47].

### 2.5. Cadmium Localization in Grey Poplar Plants

**Figure 2:**
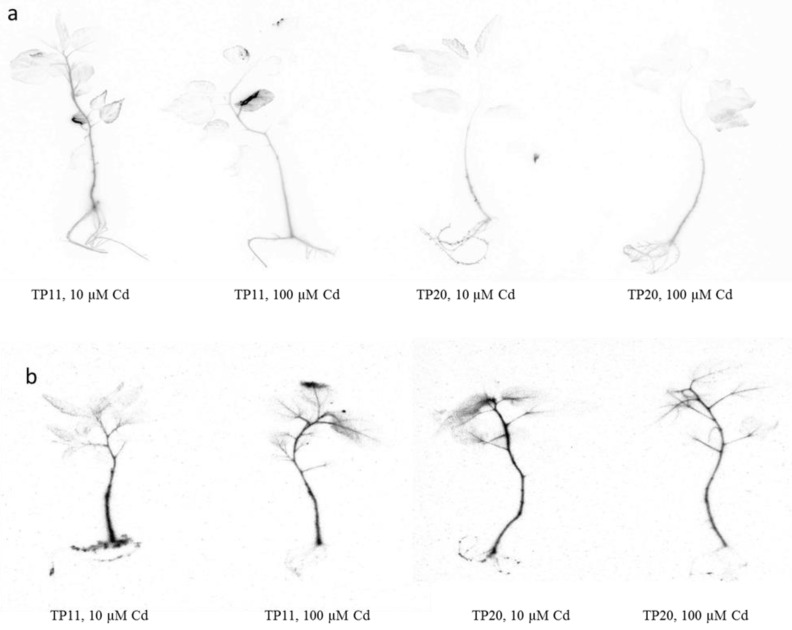
Autoradiography of hydroponically grown *Populus ×canescens* (Aiton Sm.) plants treated with Cd for 2 (**a**) and 10 (**b**) days. Radioactivity of ^109^Cd is detectable in different organs of grey poplar.

Autoradiography (Figure 2) was used to determine Cd distribution in different organs in tested grey poplar plants. Using this technique we were able to show that Cd was localized at the lower metal concentration (10 µM) mainly in the root system and stalk of both grey poplar genotypes TP11 and TP20. In plants grown on higher Cd concentration (100 µM), the Cd activity was transferred mainly into stalks and leaves (leaf veins), while in roots Cd activity decreased. This can be explained by stomatal opening and high transpiration leading to enhanced Cd transport into leaves from the veins [48]. Vollenweider et al. [49] also reported that the highest amount of Cd was located in the pectin-rich collenchyma cell walls of the veins. On the other hand, Pietrini et al. [48] showed the presence of Cd in necrotic areas of willow leaves. Cosio et al. [50] found Cd both inside the cells and in the cell walls, in the large epidermal cells but also in small epidermal cells, from which it is assumed that Cd is stored in the less metabolically active parts of leaf cells of *T. caerulescens*. The authors also report that the intensity of the darkening did not reflect the total concentration of Cd found in the leaves, therefore, it is difficult to compare autoradiography with real measured values. This confirms the comparison of autoradiography of poplar (Figure 2) with the graphs (Figure 1) in our experiment.

### 2.6. Analysis of Gene Expression Patterns

To determine how the accumulation of Cd altered the transcript levels of selected stress-response genes in actively growing grey poplar plants, qRT-PCR was conducted. The gene expression levels in two genotypes (TP11 and TP20) grown in the presence of 0 µM Cd (control), 10 µM Cd and 100 µM Cd for 2 or 10 days were detected in the roots and shoots separately (Table 5).

Chitinases belong to the glycosyl hydrolase family with a wide range of functions, including proper growth and development and resistance towards pathogens, fungi, cold stress and drought stress [51,52]. However, some chitinases are also upregulated by heavy metals [53]. The expression patterns of endochitinase 2 (LOC7470435) significantly increased in the roots of both grey poplar genotypes after treatment for 2 days with 10 µM Cd (6.85; 7.20) and 100 µM (3.36; 4.63) compared to the control (1.15; 1.03), while in the leaves, up- and downregulation was detected (Table 5). For example, the lowest transcript levels (4.3-fold) were measured in the leaves of TP20 after 2 days of 100 µM Cd treatment, whereas the highest mRNA levels (2.1-fold) were detected in the similarly treated TP11 leaves (2 days in 100 µM Cd). In contrast to our results, Gálusová et al. [54] showed that one of the tested chitinase isoforms was affected by metal stress in roots, while in leaves, the chitinases were relatively more responsive.

12-Oxophytodienoate reductases (OPRs) are a small group of flavin-dependent oxidoreductases in plants with catalytic function in the biosynthesis of jasmonic acid (JA) [55]. However, detailed information describing the mechanisms underlying JA-mediated enhanced plant Cd tolerance is rare. It has been reported that JA activates genes that might be involved in the signal transduction pathway for Cu and Cd and upregulates GSH metabolic genes [56]. Moreover, JA stimulates an increase in ROS during the first hours of exposure to excess Cu or Cd in *Arabidopsis thaliana* (L.) Heynh. [57]. Our results show that with prolonged cultivation time of grey poplar genotypes, the relative expression patterns of OPR1 increased in both plant organs. Significantly higher OPR1 transcript levels were detected in the roots of TP11 and TP20 after 10 days of Cd treatment, especially in TP11 (38.4-fold) grown under 10 µM Cd, compared to the control. As in the roots, the level of transcription in the leaves was also upregulated after 10 days of Cd treatment. The highest upregulation of ORP1 (16.4-fold) was measured in the leaves of TP20 cultivated with 10 µM Cd. Similar to our results, Liu et al. [58] showed the upregulation of OPR1 in *Arabidopsis* seedlings under Pb stress. Based on our analysis, it is conceivable that cross-talk between JA biosynthesis and the Cd defence mechanisms occurs.

Thaumatin-like proteins (TLPs) are polypeptides that share sequence similarity with thaumatin [59]. Their expression is induced by pathogen stress, but many are also inducible by other stress conditions, including heavy metal stress [60]. In the leaf apoplast of *Vigna unguiculata* (L.) Walp., Mn toxicity increased the concentration of proteins, which exhibited homology to pathogenesis-related proteins, including thaumatin-like proteins [61]. Similarly, the excess 100 µM Cd supply in our experiment caused an increase in TLP (LOC105129022) transcript levels in the roots of TP20 (7.02-fold) after 2 days of stress. We suggest that this significant change indicates a higher potential for the TP20 genotype to mediate Cd stress.

Photosystem II 10 kDa polypeptide is located at the inner grana thylakoid surface. It is well known that high Cd concentrations lead to lower values of maximum photochemical efficiency of photosystem II, which was described, for example, by Júnior et al. [62] in the study on young plants of *Virola surinamensis* (Rol. ex Rottb.) Warb. Interestingly, in our experiment, 10 days of Cd treatment did not alter the level of PSII 10 kDa polypeptide (LOC18095761) transcripts in either of the tested genotypes. A minor decrease in transcripts was observed only after 2 days of 100 µM Cd treatment in both tested genotypes. From these results, we suggest that under long-term stress, poplar plants can effectively reduce the impact of heavy metal toxicity.

Metallothioneins (MTs) are Cys-rich metal chelators. In *Vicia faba* L. transgenotes, the Arabidopsis gene *AtMT2a* was shown to play a role in Cd resistance [63]. The results of our experiment show mostly minor, non-significant changes in the level of the *MT2a* transcript. The only significant increase was found in the roots of TP11 (3.4-fold) after 10 days of 10 µM Cd treatment. Therefore, we hypothesize that *MT2a* may not participate in Cd detoxification in grey poplar.

Glutathione S-transferase expression is induced by a wide range of biotic and abiotic stresses, including pathogens, heavy metals, drought, salt or hormones [64,65,66,67]. In *Oryza sativa* L., the GST proteins might alleviate Cu toxicity by the binding of free Cu^2+^ in cells [68], which might be an important mechanism in defence towards heavy metal toxicity. Under Cd stress, the expression patterns of the 20 selected GST genes in Chinese cabbage showed differences in tissue distribution and with environmental changes [69]. However, the GST superfamily is composed of 81 genes in *Populus* [70]; thus, the mechanisms involved in the response to different stresses should be further studied. One of the classes identified was the phi class glutathione S-transferase which was found to reduce levels of oxidative damage and improved resistance to salt and drought stress [67]. Our results revealed several significant changes in GSTF4 transcript levels in response to Cd. After 10 days of Cd treatment, the GSTF4 transcript level significantly increased in the roots of both grey poplar genotypes compared to control plants and compared to the treatment for 2 days. Only in the roots of TP20 did the 2-day treatment with 100 µM Cd cause a minor, but significant, increase in GSTF4 transcript levels. Consistent with our results, Kieffer et al. [71] showed in their enzymatic assay that higher GST activity was detected in response to Cd in the roots but not in the leaves of poplar.

UDP-glucosyltransferase 74E2 (UGT74E2) is a member of the UGTs, which perturb IBA and auxin homeostasis, improve stress tolerance and regulate morphological and physiological stress adaptation mechanisms in *A. thaliana* [72]. Because Cd negatively affects auxin homeostasis [73], this gene was chosen for analysis in our study. However, our results were less consistent. The induction of transcript levels was found in the leaves of TP11 treated with 10 µM Cd for 2 days and in roots stressed with the same treatment for 10 days compared to control conditions. In contrast, transcript levels decreased in the leaves of both genotypes after 2 and 10 days of 100 µM Cd treatment.

## 3. Materials and Methods

### 3.1. Plant Material and In Vitro Culture Establishment

For this study, two individuals of *P. canescens* originating from a unique grey poplar population located in Dyjákovice village in the Czech Republic were selected as source trees. Branches were collected in March 2016 and immediately stored in a container at 4 °C. Twigs with dormant axillary buds were rinsed with running tap water for 30 min, surface disinfected with 1% sodium hypochlorite (*v*/*v*) for 20 min, 50% Korsolex plus (*v*/*v*) for 20 min, sterile distilled water for 20 min, and 1% HgCl_2_ (*w*/*v*) for 20 min and finally rinsed three times with sterile distilled water for 15 min. The sterile dormant buds were then placed into glass jars containing 50 mL modified agar MS medium [74] supplemented with 10 mg L^−1^ glutamine, 2 mg L^−1^ glycine, 0.1 mg L^−1^ indole-3-butyric acid (IBA), 0.2 mg L^−1^ 6-benzylaminopurine (BAP), 30 g L^−1^ sucrose, and 6 g L^−1^ agar at a pH of 5.8 for the induction of organogenesis. Explants were grown under controlled conditions at a photon flux density of 30 µmol m^−2^s^−1^ (16/8 day/night period) at 21 °C for 30 days. Once shoots had elongated from axillary buds, they were excised and transferred for multiplication into the same modified MS agar medium used for induction. Explants were cultured in glass jars as previously described. The shoots were subcultured every four weeks.

### 3.2. Root Induction and Acclimatization

The single grey poplar shoots were individually rooted on MS medium that did not contain any growth regulators for the induction of rhizogenesis. In total, each genotype was represented by 25 explants for the experiment. After four weeks, the rooted shoots were transferred into perlite at temperature 22 ± 2 °C for 14 days for acclimatization. High humidity was maintained by covering the pots with plastic sheets. The plants were watered with 1/10 strength MS medium. After acclimatization, fourteen-day-old poplar plantlets were treated with 10 µM or 100 µM CdCl_2_. A concentration of 100 µM Cd was chosen to provoke a fast response to stressful conditions. Untreated plants were used as a control. Samples were collected 2 and 10 days after treatment for all analyses and measurements.

### 3.3. DNA Extraction, PCR and Genotyping

DNA was extracted from 20 mg of lyophilized leaves from source trees (TP11 and TP20) using a DNeasy Plant Mini Kit (Qiagen, Germantown, MD, USA) with the protocol provided by the manufacturer. The concentration and purity of DNA was determined using a NanoPhotometer (Implen, München, Germany). DNA was stored at 4 °C.

For polymerase chain reactions (PCRs), fifteen nuclear microsatellite loci, WPMS5, WPMS15, WPMS16, WPMS18, WPMS19, WPMS20, ORPM14, ORPM16, ORPM20, ORPM30, ORPM60, ORPM127, ORPM193, ORPM220 and ORPM312, were used that were formerly described by [75,76,77,78,79]. The PCR programme, conditions and multiplex arrangement were performed according to Pokorna et al. [80]. Reactions were performed in a Veriti Thermal cycler programmed for an initial melting at 94 °C for 3 min followed by 35 cycles at 94 °C for 45 s and 55 °C for 45 s. A final extension step at 72 °C for 20 min was performed. Then, 1 µL of PCR product was mixed with 0.4 µL of Gene Scan^TM^ 600 LIZ^®^ internal size standard (Applied Biosystems, Foster City, CA, USA) and 11 µL of formamide (Hi-Di^TM^ Formamide, Foster City, CA, Applied Biosystems). After denaturation at 94 °C for 3 min and immediate chilling on ice, the products were analysed with capillary gel electrophoresis using a genetic analyser 3500 (Applied Biosystems, Foster City, CA, USA). The detection of the PCR product was enabled by the fluorescent label attached to the 5′ end of the forward primer. Allele calling was performed using GeneMapper^®^ 4.1 software provided by Applied Biosystems. Allele binning was performed manually after plotting the fragment size distribution for each locus [81].

### 3.4. RNA Extraction and cDNA Synthesis

Total RNA was extracted from mature grey poplar plants (control, 10 and 100 µM Cd), stressed roots and shoots were collected separately from three different plantlets at the end of days 2 and 10. Roots and shoots were frozen in liquid nitrogen and stored at −80 °C until analysis. RNA was extracted from homogenized plant material (approximately 100 mg of fresh weight) using the RNeasy Plant Mini Kit (Qiagen, Hilden, Germany) according to the manufacturer’s instructions. RNA concentration was measured by the MaestroNano Pro (MaestroGen, Las Vegas, NV, USA). Extracted RNA (2 µg) was reverse transcribed by M-MLV Reverse Transcriptase (Promega corporation, Madison, WI, USA), while oligodT primers were used for first-strand cDNA synthesis for each sample. According to the manufacturer´s protocol, RNA templates with primers were preincubated for 5 min at 75 °C and immediately cooled on ice. The RT-PCR premix was then added, and the transcription reaction was run at 42 °C/60 min, 70 °C/5 min, and 10 °C until further manipulation.

### 3.5. Gene Expression Analysis

The quantitative real-time RT-PCR analyses were carried out using GoTaq qPCR Master Mix (Promega Corporation, Madison, WI, USA) mix in a LightCycler 96 (Roche, Mannheim, Germany). The analyses were performed using two reference genes and seven target genes reported previously for poplar trees (Table 6). The primers were designed using Primer3 software (Rozen and Skaletsky 2000, Boston, MA, USA) according to the following parameters: 100–200 bp product size, 19–22 bp primer length, 58–60 °C melting temperature (Tm) and 45–55% GC content. The thermal cycling conditions were assessed 120 s at 95 °C for preincubation, followed by amplification for 45 cycles of 95 °C for 10 s, 60 °C for 10 s, 72 °C for 10 s, melting curve was assessed at 95 °C for 10 s, followed by 65 °C for 60 s and 97 °C for 1 s. Analysis of all samples was performed with three technical replicates. Quantifications were performed according to the ∆Ct method, and the relative gene expression levels were normalized by the expression levels of the 18 S ribosomal RNA and elongation factor 1-α genes, as internal standards [82].

### 3.6. Measurement of Cd and Selected Element Contents

The content of Cd and selected minerals (K, Ca, Mg, Zn) in the roots and shoots of grey poplar samples was determined using ICP-OES (Inductively Coupled Plasma Optical Emission Spectrometry, Varian 725-ES, Agilent Technologies, Santa Clara, USA). Dried material was milled to fine powder, whereas each sample (100 mg fresh weight) was mineralized with 5 mL of nitric acid [67% HNO_3_ (*w*/*v*)] and 1 mL of hydrogen peroxide [30% H_2_O_2_ (*w*/*v*)] in a microwave digestion system (Speedwave4, Berghof Products Instruments, Eningen, Germany) at 200 °C for 15 min. Cooled samples were diluted with distilled water, and the element content was determined by ICP-OES (Varian 725-ES, Agilent Technologies, Santa Clara, USA). Three biological replicates of control and Cd-treated plants were analysed in two individual sets of experiments.

### 3.7. Chlorophyll Measurement

Fresh shoot tissues (100 mg) were extracted in 80% acetone (*w*/*v* 1 g FW 20 mL^−1^) and centrifuged for 14,000 rcf for 5 min at 4 °C. The supernatant was separated, and 0.2 mL of the supernatant was mixed with 0.8 mL of acetone. The solution mixture was analysed for chlorophyll *a*, chlorophyll *b* and chlorophyll *a + b* contents by recording the absorbance at 663 (chlorophyll *a*) and 647 (chlorophyll *b*) using a UV-VIS spectrophotometer (VIS-7236, Rayleigh, Beijing Beifen-Ruili Analytical Instrument, Beijing, China). The contents of photosynthetic pigments were calculated according to [89].

### 3.8. Autoradiography Method

The grey poplar plantlets (genotypes TP11 and TP20) were transferred from in vitro conditions to the greenhouse and cultivated in the modified Hoagland medium [90]. The hydroponic medium with a pH adjusted to 5.0 contained 4 mM CaCl_2_, 2 mM K_2_SO_4_, 2 mM NH_4_NO_3_, 2 mM NaH_2_PO_4_, 1.5 mM MgSO_4_, 4 mM NaNO_3_, 4 mM NH_4_Cl, 0.2 mM FeSO_4_, 138.8 µM H_3_BO_3_, 20.8 µM MnSO_4_, 2.3 µM ZnSO_4_, 3.3 µM CuSO_4_ and 0.2 µM Na_2_MoO_4_ (PENTA Ltd., Prague, Czech Republic). The plants were maintained at 23 °C with a relative humidity of approximately 60% and were irradiated with 16 h of light (average irradiation of 72 µmol m^−2^s^−1^—at the plant surface, with horizontal differences in irradiation less than 20%, sodium discharge lamps at 400 W, Thorn Radbay, Durhamgate, UK). Two-week-old plants were used for the experiments.

For the experiment the modified Hoagland medium contain 0.01 or 0.1 mM Cd(NO_3_)_2_ × 4H_2_O (PENTA Ltd., Czech Republic) and volume activity of 6.517 MBq/L of ^109^Cd as CdCl_2_ in HCl water solution (3g HCl/L) (specific activity 4.591 MBq/g, EUROSTANDARD CZ Ltd., Prague, Czech Republic) was used. After exposure (2 and 10 days), the roots of the plants were washed with distilled water, EDTA (concentration 0.1 mM) and distilled water and then the plants were pressed and dried (between two filter papers, 25 °C, ca 1 week). The pressed plants were transferred to an Exposure Cassette (Amersham Biosciences, Little Chalfont, UK) (24 × 30 cm) and put on the Kodak Storage Phosphor Screen S 230. The screen was exposed during 120 h (2 days Cd treatment) or 24 h (10 days Cd treatment) and scanned by Typhoon Imager (Amersham Biosciences, Little Chalfont, UK). The data were visualized by the program Image Quant TL.

### 3.9. Data Analysis

All analyses were performed in three biological replicates using two-way ANOVA and Tukey’s test (Minitab) to evaluate the significant differences (*p* < 0.05) among the Cd treatments (0 µM, 10 µM and 100 µM).

## 4. Conclusions

To reveal the genetic differentiation between two selected grey poplar genotypes, microsatellite analysis was performed. Considering the toxicity of Cd and the effects of Cd on plant growth, the present data demonstrated that grey poplar cultures are highly tolerant to metal stress. However, the chlorophyll content in the shoots significantly decreased in Cd-treated plants. According to the mineral concentration, the two poplar genotypes, TP11 and TP20, showed variation in mineral uptake after Cd treatment. Cadmium accumulation was higher in the roots than in the shoots, and after 10 days of exposure to 10 µM Cd, genotype TP20 translocated comparable amounts of Cd to the roots and shoots. This accumulation was reflected by a TF value greater than 1, which indicates that Cd tolerance in genotype TP20 was greater than that in TP11 under the current experimental conditions. However, autoradiography did not confirm differences in Cd accumulation between both tested cultivars. According to the expression of stress-response genes, the most significant change was observed in the amount of endochitinase 2, 12-oxophytodienoate reductase 1 and phi class glutathione S-transferase transcripts.

The present data demonstrated that in vitro cultures of cuttings are useful for assessing the ability of different poplar genotypes to tolerate Cd and for assessing the mechanisms underlying Cd tolerance. Furthermore, studies on metal tolerance based on the association of physiological and molecular data represent an initial effective method for field trials on metal-polluted soils.

## Figures and Tables

**Table 1:** Summary of the allele sizes obtained from the SSR analysis.

SSR	Allele Size	
	TP11	TP20
WPMS5	298	276/310
WPMS15	189/192	189/192
WPMS16	165/171	165/171
WPMS18	222/228	219/222
WPMS19	195/204	186
WPMS20	178	178
ORPM14	141	141
ORPM16	223	223
ORPM20	180/196	197/201
ORPM30	221/229	207/219
ORPM60	200/206	206
ORPM127	189	189
ORPM193	175/190	175/190
ORPM220	217/229	217
ORPM312	195	195

**Table 2:** Root length (cm), root weight (mg), shoot weight (mg) and chlorophyll content (mg g^−1^ FW) in two different cultivars of grey poplar (*Populus ×canescens* Aiton Sm.) plants after 2 and 10 days of growth in the presence of different concentrations of Cd. Data are presented as the mean ± SD (n = 3). Values followed by the same letter(s) within the same column are not significantly different according to Tukey’s test (*p* ˂ 0.05).

		Cd (µM)	Root Length	Root Weight	Shoot Weight	Chl *a*	Chl *b*	Chl *a + b*
2 days	TP11	0	10.7 ± 2.0 ^d^	47.3 ± 6.6 ^e^	196.7 ± 59 ^bc^	2.49 ± 0.3 ^ab^	0.89 ± 0.1 ^ab^	3.38 ± 0.4 ^ab^
		10	14.7 ± 0.8 ^abc^	75.0 ± 3.6 ^bcd^	255.7 ± 49 ^bc^	1.02 ± 0.2 ^ef^	0.24 ± 0.1 ^cd^	1.26 ± 0.2 ^de^
		100	15.3 ± 1.3 ^abc^	85.3 ± 4.2 ^abc^	226.0 ± 56 ^bc^	0.62 ± 0.2 ^f^	0.21 ± 0.1 ^d^	0.83 ± 0.2 ^e^
	TP20	0	10.8 ± 2.3 ^d^	52.3 ± 5.8 ^e^	199.0 ± 63 ^bc^	2.27 ± 0.2 ^b^	0.65 ± 0.1 ^abcd^	2.92 ± 0.3 ^b^
		10	13.6 ± 0.5 ^bcd^	71.3 ± 5.5 ^cd^	157.7 ± 39 ^c^	2.10 ± 0.1 ^bc^	0.43 ± 0.2 ^bcd^	2.52 ± 0.1 ^bc^
		100	15.1 ± 0.8 ^abc^	84.3 ± 5.5 ^abc^	151.0 ± 45 ^c^	1.47 ± 0.3 ^cde^	0.33 ± 0.1 ^bcd^	1.83 ± 0.3 ^cd^
10 days	TP11	0	12.2 ± 1.0 ^cd^	79.3 ± 5.1 ^abcd^	440.7 ± 39 ^a^	3.07 ± 0.5 ^a^	0.84 ± 0.1 ^ab^	3.91 ± 0.3 ^a^
		10	15.5 ± 1.2 ^abc^	89.7 ± 6.7 ^ab^	448.7 ± 45 ^a^	2.17 ± 0.1 ^bc^	0.78 ± 0.1 ^abc^	2.95 ± 0.2 ^b^
		100	15.8 ± 1.7 ^ab^	93.0 ± 7.9 ^a^	301.4 ± 40 ^abc^	1.08 ± 0.2 ^cde^	1.08 ± 0.1 ^a^	2.55 ± 0.2 ^bc^
	TP20	0	12.3 ± 0.5 ^cd^	62.3 ± 4.9 ^de^	328.3 ± 69 ^ab^	3.09 ± 0.3 ^a^	0.81 ± 0.2 ^abc^	3.90 ± 0.4 ^a^
		10	16.0 ± 0.4 ^ab^	77.1 ± 7.8 ^abcd^	291.8 ± 91 ^abc^	2.03 ± 0.3 ^bcd^	0.69 ± 0.3 ^abcd^	2.72 ± 0.6 ^bc^
		100	17.1 ± 0.3 ^a^	81.3 ± 7.7 ^abc^	274.1 ± 43 ^bc^	1.40 ± 0.1 ^de^	0.52 ± 0.2 ^abcd^	1.91 ± 0.1 ^cd^

**Table 3:** Contents of K, Ca, Mg, and Zn (g kg^−1^ DW) in the roots and shoots of *Populus ×canescens* (Aiton Sm.) genotypes TP11 and TP20 grown in perlite for 2 or 10 days with or without Cd (0 µM, 10 µM and 100 µM). Data are presented as the mean ± SD (n = 3). Values followed by the same letter(s) within the same column are not significantly different according to Tukey’s test (*p* ˂ 0.05).

			K Content (g kg^−1^)		Ca Content (g kg^−1^)		Mg Content (g kg^−1^)		Zn Content (g kg^−1^)	
			Roots	Shoots	Roots	Shoots	Roots	Shoots	Roots	Shoots
2 days	TP11	Cont	13.24 ± 1.93 ^hij^	30.19 ± 1.84 ^a^	5.05 ± 0.21 ^fgh^	6.60 ± 1.11 ^cde^	1.64 ± 0.15 ^bcd^	1.50 ± 0.11 ^cd^	0.22 ± 0.05 ^ab^	0.16 ± 0.06 ^abcdef^
		Cd10	10.40 ± 0.87 ^j^	23.57 ± 2.75 ^bcde^	4.04 ± 0.31 ^h^	5.93 ± 0.70 ^def^	1.42 ± 0.26 ^cd^	1.64 ± 0.18 ^bcd^	0.20 ± 0.04 ^abc^	0.17 ± 0.01 ^abcde^
		Cd100	12.50 ± 1.19 ^hij^	24.29 ± 2.58 ^bcde^	4.69 ± 0.36 ^fgh^	8.54 ± 0.44 ^ab^	2.02 ± 0.43 ^abcd^	2.41 ± 0.52 ^abc^	0.25 ± 0.04 ^a^	0.17 ± 0.03 ^abcd^
	TP20	Cont	12.22 ± 2.68 ^hij^	21.24 ± 1.60 ^cdef^	5.03 ± 0.91 ^gh^	7.07 ± 0.42 ^abcd^	1.53 ± 0.51 ^bcd^	1.85 ± 0.99 ^abcd^	0.14 ± 0.02 ^bcdef^	0.08 ± 0.00 ^f^
		Cd10	16.03 ± 3.12 ^fgh^	22.81 ± 2.02 ^bcdef^	4.39 ± 0.58 ^gh^	6.86 ± 0.38 ^bcd^	2.22 ± 0.11 ^abcd^	1.95 ± 0.18 ^abcd^	0.17 ± 0.07 ^abcdef^	0.08 ± 0.03 ^ef^
		Cd100	16.25 ± 0.38 ^ghi^	25.60 ± 1.52 ^abcd^	4.75 ± 1.04 ^gh^	6.68 ± 0.84 ^abcd^	2.83 ± 0.20 ^a^	1.94 ± 0.01 ^abcd^	0.18 ± 0.02 ^abcd^	0.09 ± 0.03 ^def^
10 days	TP11	Cont	11.70 ± 0.98 ^ij^	27.43 ± 2.47 ^abc^	5.04 ± 1.03 ^gh^	6.64 ± 0.61 ^abcd^	1.67 ± 0.00 ^bcd^	1.73 ± 0.51 ^abcd^	0.21 ± 0.03 ^abc^	0.14 ± 0.01 ^bcdef^
		Cd10	14.76 ± 2.31 ^hij^	27.36 ± 1.73 ^abc^	3.78 ± 0.31 ^h^	5.49 ± 0.96 ^defg^	1.13 ± 0.22 ^d^	1.83 ± 0.45 ^abcd^	0.14 ± 0.01 ^bcdef^	0.21 ± 0.04 ^abc^
		Cd100	20.35 ± 1.33 ^defg^	26.88 ± 2.84 ^ab^	4.41 ± 0.72 ^gh^	8.13 ± 0.81 ^a^	2.02 ± 0.04 ^abcd^	2.64 ± 0.77 ^ab^	0.16 ± 0.03 ^bcdef^	0.22 ± 0.03 ^ab^
	TP20	Cont	9.96 ± 1.54 ^j^	19.40 ± 0.59 ^defg^	4.66 ± 0.63 ^fgh^	6.45 ± 1.06 ^cd^	2.14 ± 0.12 ^abcd^	1.64 ± 0.25 ^bcd^	0.18 ± 0.04 ^abcd^	0.08 ± 0.00 ^f^
		Cd10	15.26 ± 2.72 ^hij^	26.46 ± 2.00 ^abcd^	3.70 ± 5.60 ^h^	6.30 ± 1.04 ^cd^	1.13 ± 0.12 ^d^	1.73 ± 0.31 ^abcd^	0.20 ± 0.02 ^abc^	0.13 ± 0.03 ^bcdef^
		Cd100	14.00 ± 3.49 ^hij^	26.81 ± 2.10 ^ab^	5.00 ± 0.51 ^fgh^	7.98 ± 0.51 ^abc^	1.93 ± 0.13 ^abcd^	2.10 ± 0.18 ^abcd^	0.13 ± 0.03 ^cdef^	0.15 ± 0.03 ^bcdef^

**Table 4:** Translocation factor [TF = C_aerial_/C_root_] in *Populus ×canescens* (Aiton Sm.) genotypes TP11 and TP20 grown in perlite for 2 or 10 days with or without Cd.

Translocation Factor (TF)		
**2 days**	**TP11**	**TP20**
Cd 10	0.34	0.54
Cd 100	0.27	0.42
**10 days**		
Cd 10	0.61	1.06
Cd 100	0.57	0.63

**Table 5:** Relative gene expression values obtained by semi-quantitative real time RT–PCR for seven candidate genes. The analysis was performed on roots and shoots of *Populus ×canescens* (Aiton Sm.) genotypes TP11 and TP20 grown in perlite for 2 or 10 days with or without Cd. Data are presented as the mean ± SD (n = 3). Values for each single gene followed by the same letter(s) are not significantly different according to Tukey’s test (*p* < 0.05).

	2 Days		10 Days	
	Root	Shoot	Root	Shoot
Endochitinase 2				
TP11				
Control	1.15 ± 0.7 ^cde^	0.97 ± 0.5 ^cde^	1.05 ± 0.2 ^cde^	0.90 ± 0.2 ^cde^
10 Cd	6.85 ± 0.8 ^a^	1.18 ± 0.2 ^cde^	1.83 ± 0.2 ^c^	0.47 ± 0.1 ^de^
100 Cd	3.36 ± 1.1 ^b^	1.99 ± 0.3 ^c^	1.55 ± 0.3 ^cd^	0.49 ± 0.2 ^de^
TP20				
Control	1.03 ± 0.1 ^cde^	0.91 ± 0.2 ^cde^	1.03 ± 0.1 ^cde^	0.90 ± 0.1 ^cde^
10 Cd	7.20 ± 0.9 ^a^	1.60 ± 0.3 ^cd^	1.40 ± 0.3 ^cde^	0.72 ± 0.2 ^cde^
100 Cd	4.63 ± 0.5 ^b^	0.21 ± 0.1 ^e^	1.77 ± 0.4 ^cd^	0.93 ± 0.2 ^cde^
12-oxophytodienoate reductase 1				
TP11				
Control	0.55 ± 0.1 ^g^	1.71 ± 0.9 ^fg^	0.92 ± 0.4 ^g^	1.56 ± 0.3 ^fg^
10 Cd	1.95 ± 0.1 ^fg^	6.09 ± 1.8 ^defg^	35.36 ± 8.4 ^a^	13.89 ± 1.7 ^c^
100 Cd	4.83 ± 1.8 ^defg^	2.10 ± 0.1 ^fg^	24.26 ± 3.9 ^b^	8.00 ± 1.1 ^cdef^
TP20				
Control	0.99 ± 0.4 ^g^	1.60 ± 0.6 ^fg^	0.87 ± 0.1 ^g^	1.59 ± 0.5 ^fg^
10 Cd	3.00 ± 0.7 ^efg^	0.93 ± 0.1 ^g^	6.57 ± 1.5 ^defg^	26.03 ± 0.9 ^b^
100 Cd	9.34 ± 0.9 ^cde^	1.97 ± 0.3 ^fg^	9.87 ± 1.9 ^cd^	10.24 ± 0.9 ^cd^
thaumatin-like protein				
TP11				
Control	0.95 ± 0.4 ^b^	1.79 ± 0.2 ^b^	0.97 ± 0.5 ^b^	2.25 ± 1.5 ^b^
10 Cd	0.25 ± 0.1 ^b^	0.25 ± 0.2 ^b^	1.32 ± 0.4 ^b^	1.24 ± 1.0 ^b^
100 Cd	1.35 ± 0.3 ^b^	1.75 ± 1.4 ^b^	0.95 ± 0.2 ^b^	1.36 ± 0.6 ^b^
TP20				
Control	0.90 ± 0.1 ^b^	1.85 ± 0.6 ^b^	0.91 ± 0.2 ^b^	1.81 ± 0.3 ^b^
10 Cd	0.47 ± 0.2 ^b^	2.24 ± 0.9 ^b^	0.92 ± 0.3 ^b^	1.56 ± 0.8 ^b^
100 Cd	6.32 ± 1.2 ^a^	0.29 ± 0.1 ^b^	1.20 ± 0.6 ^b^	1.54 ± 0.6 ^b^
photosystem II 10 kDa polypeptide				
TP11				
Control	-	1.42 ± 1.1 ^abc^	-	1.23 ± 0.5 ^abc^
10 Cd	-	1.88 ± 0.3 ^ab^	-	2.25 ± 0.2 ^ab^
100 Cd	-	0.73 ± 0.1 ^bc^	-	2.43 ± 1.1 ^a^
TP20				
Control	-	1.25 ± 0.6 ^abc^	-	1.17 ± 0.1 ^abc^
10 Cd	-	0.78 ± 0.1 ^bc^	-	2.34 ± 0.3 ^a^
100 Cd	-	0.04 ± 0.0 ^c^	-	1.20 ± 0.1 ^abc^
Metallothionein 2a				
TP11				
Control	2.86 ± 1.2 ^bcdef^	1.10 ± 0.4 ^defgh^	2.84 ± 1.0 ^bcdefg^	1.11 ± 0.5 ^defgh^
10 Cd	0.74 ± 0.6 ^efgh^	1.23 ± 0.2 ^cdefgh^	9.68 ± 1.8 ^a^	1.42 ± 0.3 ^bcdefgh^
100 Cd	3.50 ± 1.3 ^b^	0.77 ± 0.2 ^defgh^	2.98 ± 0.7 ^bcd^	1.05 ± 0.6 ^defgh^
TP20				
Control	2.74 ± 0.5 ^bcdefg^	1.14 ± 0.6 ^defgh^	2.90 ± 1.3 ^bcde^	1.04 ± 0.1 ^defgh^
10 Cd	0.84 ± 0.2 ^defgh^	0.63 ± 0.2 ^gh^	3.43 ± 0.5 ^bc^	1.23 ± 0.1 ^cdefgh^
100 Cd	1.66 ± 0.1 ^bcdefgh^	0.07 ± 0.0 ^h^	2.10 ± 0.1 ^bcdefgh^	0.66 ± 0.0 ^fgh^
phi class glutathione S-transferase				
TP11				
Control	1.90 ± 0.7 ^d^	1.04 ± 0.1 ^d^	1.91 ± 0.8 ^d^	1.24 ± 0.7 ^d^
10 Cd	2.97 ± 0.4 ^d^	3.92 ± 0.1 ^d^	20.43 ± 0.9 ^a^	0.94 ± 0.2 ^d^
100 Cd	1.33 ± 0.8 ^d^	2.75 ± 0.2 ^d^	15.65 ± 1.6 ^b^	0.05 ± 0.4 ^d^
TP20				
Control	1.80 ± 0.2 ^d^	1.21 ± 0.9 ^d^	1.82 ± 0.4 ^d^	1.12 ± 0.5 ^d^
10 Cd	2.89 ± 0.8 ^d^	2.97 ± 0.3 ^d^	16.03 ± 1.3 ^b^	2.59 ± 1.5 ^d^
100 Cd	8.50 ± 4.9 ^c^	2.21 ± 0.0 ^d^	17.50 ± 1.3 ^ab^	1.99 ± 0.9 ^d^
Uridine diphosphate glycosyltransferase 74E2				
TP11				
Control	1.02 ± 0.1 ^defg^	1.72 ± 0.1 ^cdef^	1.04 ± 0.3 ^defg^	1.76 ± 0.5 ^cdef^
10 Cd	1.13 ± 0.4 ^defg^	3.73 ± 0.4 ^ab^	4.98 ± 1.2 ^a^	0.81 ± 0.2 ^efg^
100 Cd	1.55 ± 0.2 ^cdefg^	1.33 ± 0.3 ^cdefg^	2.32 ± 0.5 ^cd^	0.33 ± 0.1 ^g^
TP20				
Control	1.01 ± 0.1 ^defg^	1.81 ± 0.7 ^cde^	1.01 ± 0.1 ^defg^	1.73 ± 0.2 ^cdef^
10 Cd	1.05 ± 0.2 ^defg^	1.17 ± 0.3 ^defg^	2.56 ± 0.8 ^bc^	2.60 ± 0.4 ^bc^
100 Cd	1.36 ± 0.5 ^cdefg^	0.45 ± 0.1 ^fg^	1.37 ± 0.2 ^cdefg^	1.39 ± 0.2 ^cdefg^

**Table 6:** The gene name, accession number, description, primer sequence and reference.

Gene	Accession Number	Gene Description	Primer Sequence (5′–3′)	Reference
LOC112328551	XR_002983567	18S Ribosomal RNA	F: AGAAACGGCTACCACATCCAA R: CCAGACTTGCCCTCCAATGG	[83]
LOC18109220	EF147878.1	Elongation factor 1-α	F: CCACACCTGTCACATTGCTG R: ACCAGCATCACCGTTCTTCAG	[84]
LOC7470435	XM_002306184.3	Endochitinase 2	F: TACGGGCAATGTGGAAAAGC R: ATTGTGGCATGAGGGCTTTG	[85]
OPR1	NM_106318.4	12-Oxophytodienoate reductase 1	F: CGGACAAGCAGGAGACTCAAA R: CCACCGTCTTCATTCTTGGC	[83]
UGT74E2	NM_100448.4	Uridine diphosphate glycosyltransferase 74E2	F: CACAAATCCGTGGGATGCTT R: TCTGTCCACTGTGGCATTGC	[83]
LOC105129022	XM_011030918	Thaumatin-like protein	F: ACCACACAAGCACGCATTTG R: TGAACCATAGCCTTGGCATG	[85]
LOC18095761	18095761	Photosystem II 10 kDa polypeptide	F: ATGGTGCTAATGTGGATGGC R: AACAGCCCAGATTAGCAAGC	[86]
MT2a	AY594297.1	Metallothionein 2a	F: ATCATCGCATCGACGGATTG R: CCAGAGCTGCAAATCCAAGAAG	[87]
GSTF4	GQ377243.1	Phi class glutathione S-transferase	F: CTTAGCCTCGTTTTCCTCCA R: CTTAGCCTCGTTTTCCTCCA	[88]
